# Thermoelectric Freeze-Casting of Biopolymer Blends: Fabrication and Characterization of Large-Size Scaffolds for Nerve Tissue Engineering Applications

**DOI:** 10.3390/jfb14060330

**Published:** 2023-06-20

**Authors:** Vincent Monfette, William Choinière, Catherine Godbout-Lavoie, Samuel Pelletier, Ève Langelier, Marc-Antoine Lauzon

**Affiliations:** 1 Department of Chemical Engineering and Biotechnological of Engineering, Faculty of Engineering, Université de Sherbrooke, Sherbrooke, QC J1K 2R1, Canada; vincent.monfette@usherbrooke.ca (V.M.); william.choiniere@usherbrooke.ca (W.C.); catherine.godbout-lavoie@usherbrooke.ca (C.G.-L.); 2 Department of Electrical Engineering and Informatics Engineering, Faculty of Engineering, Université de Sherbrooke, Sherbrooke, QC J1K 2R1, Canada; samuel.pelletier@usherbrooke.ca; 3 Department of Mechanical Engineering, Faculty of Engineering, Université de Sherbrooke, Sherbrooke, QC J1K 2R1, Canada; eve.langelier@usherbrooke.ca; 4 Research Center on Aging, CIUSSS de l’ESTRIE-CHUS, Sherbrooke, QC J1H 4C4, Canada

**Keywords:** biomaterials, Schwann cells, peripheral nerve injury, nerve guidance conduit, mechanical traction tests

## Abstract

Peripheral nerve injuries (PNIs) are detrimental to the quality of life of affected individuals. Patients are often left with life-long ailments that affect them physically and psychologically. Autologous nerve transplant is still the gold standard treatment for PNIs despite limited donor site and partial recovery of nerve functions. Nerve guidance conduits are used as a nerve graft substitute and are efficient for the repair of small nerve gaps but require further improvement for repairs exceeding 30 mm. Freeze-casting is an interesting fabrication method for the conception of scaffolds meant for nerve tissue engineering since the microstructure obtained comprises highly aligned micro-channels. The present work focuses on the fabrication and characterization of large scaffolds (35 mm length, 5 mm diameter) made of collagen/chitosan blends by freeze-casting via thermoelectric effect instead of traditional freezing solvents. As a freeze-casting microstructure reference, scaffolds made from pure collagen were used for comparison. Scaffolds were covalently crosslinked for better performance under load and laminins were further added to enhance cell interactions. Microstructural features of lamellar pores display an average aspect ratio of 0.67 ± 0.2 for all compositions. Longitudinally aligned micro-channels are reported as well as enhanced mechanical properties in traction under physiological-like conditions (37 °C, pH = 7.4) resulting from crosslinking treatment. Cell viability assays using a rat Schwann cell line derived from sciatic nerve (S16) indicate that scaffold cytocompatibility is similar between scaffolds made from collagen only and scaffolds made from collagen/chitosan blend with high collagen content. These results confirm that freeze-casting via thermoelectric effect is a reliable manufacturing strategy for the fabrication of biopolymer scaffolds for future peripheral nerve repair applications.

## 1. Introduction

The peripheral nervous system (PNS) relays sensory and motor information from the brain and the spinal cord to the rest of the body. Consequently, injuries affecting the PNS are debilitating in nature and can lead to permanent disability or poor limb functions as well as chronic pain [1,2]. Worldwide, about 1 in 1000 people sustain a PNI annually and most of the afflicted individuals are part of the current workforce, occasioning socio-economic consequences [3,4]. For civilians, the most common causes of PNIs are motor vehicle collisions [3,5,6], lacerations caused by sharp objects, stabbing [7], and crushing injuries [8]. Armed conflicts are also responsible for a significant number of PNIs, which result from blast and gunshot injuries [9,10,11]. Due to advancements in battlefield healthcare, medical evacuation, and the use of body armor, an even larger number of warfighters are surviving and returning with PNIs [12,13,14,15].

The microsurgical procedure is mandatory for the repair of nerve transections, where the nerve end-to-end coaptations must be tensionless to prevent ischemia, or else nerve grafts must be used to bridge gaps [16,17,18,19]. Currently, the autograft (autologous nerve transplantation) is the gold standard treatment for severe injuries despite limited donor sites, possible mismatch, poor outcomes, and additional surgical costs. The next best solution consists of bridging nerve gaps using a nerve guidance conduit (NGC), which is made from natural or synthetic biopolymers and designed to support nerve regeneration. Currently, NGCs are sufficient for the reparation of small nerve gaps (<30 mm) but less effective than the autograft for bigger repairs [20,21,22,23], and their mechanical properties underperform compared to the natural nerve [24,25,26]. At the moment, experimental and clinical data regarding nerve reconstruction on gaps exceeding 30 mm are scarce, while most scientific studies use small-size NGCs to conduct experimental tests [27,28].

The native peripheral nerve is composed of longitudinally oriented basal lamina tubes, filled with Schwann cells surrounding individual axons. During regeneration, axons will disperse in the absence of structural support, thus requiring guidance to reconnect to their original innervation target [29]. Dispersion is also observed in single-channel NGCs and can be further limited by opting for a multichannel structure [30]. Current FDA-approved NGCs made from biopolymers are mostly semi-permeable hollow tubes with only one product (i.e., Neuragen 3D) comprising a multichannel structure [31,32]. Unidirectional freeze-casting, which relies on a temperature gradient to freeze a biopolymer mixture in a controlled way, and therefore obtains a certain crystal formation, can be used as an advanced fabrication technique to obtain highly porous and anisotropic scaffolds [33]. Using this technique, porous micro-channels mimicking the physiology of the natural nerve endoneurium can be obtained [33,34]. Previous work using freeze-casting to shape biomaterials used freezing solvent in their system which can be replaced by a thermoelectric module [35,36,37]. Unidirectional thermoelectric freeze-casting systems have yet to be tested with popular biopolymers to evaluate their efficacy at producing the desired microstructure. The resulting anisotropic property of scaffolds obtained by unidirectional freeze-casting is important to recreate the mechanical behavior of natural nerves. Current studies covering the characterization of NGCs in terms of mechanical properties are scarce [38]. Moreover, only a few publications report mechanical evaluation of large-size scaffolds (≥30 mm) [39,40] which should be conducted under physiological-like conditions (37 °C, hydrated in PBS) [41,42].

To date, there are no FDA (US Food and Drug Administration)-approved NGCs recommended for the reparation of large nerve gaps (>30 mm) [28]. Most commercial NGCs made from natural biopolymers are made using type I collagen (coll) [27]. Some other products are made of poly (dl-lactide-ε-caprolactone) (PCL) or chitosan (chit), which are the most frequently used and approved biomaterials including collagen [27]. Being the most abundant structural protein of the body, collagen is extensively used in tissue engineering applications [43] and can be chemically treated to compensate for its limited/lacking mechanical strength [44]. Recent investigations support chitosan as a promising biomaterial for peripheral nerve regeneration for defects with sizes ranging from 5 to 30 mm [45]. Chitosan has also been shown to promote cell adhesion and proliferation [46], while PCL offers poor cell-adhesion due to its hydrophobic nature and usually requires further surface modification [47]. Other work reported the potential of collagen–chitosan biopolymer blends as new tailorable/adaptable materials for tissue engineering applications [44,48]. Even though previous work showed good regeneration outcomes in vivo using an NGC based on a collagen/chitosan blend, research on the characterization of scaffolds with continuous micro-channels made from various collagen–chitosan blends for the reparation of large nerve gaps is limited.

To support regeneration, NGCs should also have pre-seeded Schwann cells [49]. Schwann cells are required for nerve repair since they play multiple roles during the regeneration process [50,51]. Proliferation and migration are limited in scaffolds, partially due to limited scaffold interactions. Therefore, laminins, which are proteins from the extracellular matrix (ECM), can be added to synthetic scaffolds to promote attachment and proliferation of Schwann cells. Laminins are also required for Schwann cell differentiation, axon myelination, and regeneration in the peripheral nerve [52,53].

In this study, a custom-made unidirectional freeze-casting system using the thermoelectric effect, instead of traditional freezing solvent [33,54,55,56,57] approaches, was used to fabricate NGC-sized cylindrical scaffolds (35 mm long, 5 mm diameter) made of biopolymer blends of different coll/chit ratios. The resulting scaffolds were then crosslinked and microstructural features as well as mechanical properties and performance under physiological conditions were assessed. Finally, these scaffolds were tested as potential cell culture scaffolds with glial cells.

## 2. Materials and Methods

### 2.1. Materials

Purified type I collagen from bovine Achilles tendon was purchased from MyBioSource (San Diego, CA, USA). Medium molecular weight chitosan (75–85% deacetylated), mouse laminins (L2020), EDC (*N*-(3-dimethylaminopropyl)-*N*′-ethylcarbodiimide) (purity ≥ 97%), NHS (*N*-hydroxysuccinimide) (purity ≥ 98%), TPP (sodium triphosphate pentabasic) (purity ≥ 98%), poly-l-lysine hydrobromide (PLL), Triton^TM^ X-100, and Normal Goat Serum (NGS) were purchased from Millipore-Sigma (Oakville, ON, Canada). Schwann cells (S16 line, CRL-2941^TM^) were obtained from ATCC, and fetal bovine serum (FBS) from Wisent Bioproducts. Dulbecco’s modified Eagle’s medium (DMEM, high glucose, GlutaMAX supplement, pyruvate), penicillin-streptomycin (Pen Strep), alamarBlue^TM^ assay reagent, Hoechst 33342, and Live/Dead^TM^ Viability/Cytotoxicity Kit (Invitrogen, Waltham, MA, USA) reagents were purchased from Life Technologies (Burlington, ON, Canada). Primary and secondary antibodies (Cell Signalling Technology Inc., Danvers, MA, USA): S100β (E7C3A) Rabbit mAb #90393 and Anti-rabbit IgG (H + L), F(ab′)2 Fragment (Alexa Fluor^®^ 488 Conjugate Green) #4412 were purchased from New England Biolabs Ltd. (Whitby, ON, Canada).

### 2.2. Biopolymers Preparation

For the collagen (coll), a 1% (*w*/*w*) suspension was prepared by adding insoluble collagen (dehydrate flakes) from bovine Achilles tendon (MBS634790, MyBioSource) to 3.8% (*w*/*v*) acetic acid (pH 3.20). The mixture was then refrigerated overnight to allow the collagen fiber to swell and then homogenized at 13,500 rpm with an overhead homogenizer (LabGEN 125, Cole-Parmer Canada Inc., Quebec, QC, Canada) for 30 min in an ice bath. Harder-to-break collagen fibers were broken down by driving the homogenizer manually against the fibers. The collagen suspension was then centrifuged at 1250 RCF at 4 °C for 8 min to remove the bigger air bubbles. The suspension was then degassed in a vacuum chamber for 40 min at −70 kPa and finally centrifuged again at 1250 RCF at 4 °C for 8 min to remove the remaining air bubbles. For the biopolymer blends, a 1% (*w*/*w*) chitosan (chit) (medium molecular weight, 75–85% deacetylated, Millipore-Sigma, Oakville, ON, Canada) solution was made using 2% (*w*/*v*) acetic acid. The same 1% (*w*/*v*) collagen suspension prepared previously was combined with the 1% (*w*/*v*) chitosan solution to obtain coll/chit weight ratios of 60/40, 80/20, and 90/10. Each biopolymer blend was then homogenized at 13,500 rpm for 10 min in an ice bath and then centrifuged at 1250 RCF at 4 °C for 5 min, degassed under vacuum for 20 min, and finally centrifuged again at 1250 RCF at 4 °C for 5 min.

### 2.3. Scaffold Preparation for Thermoelectric-Based Unidirectional Freeze-Casting

**Figure 1:**
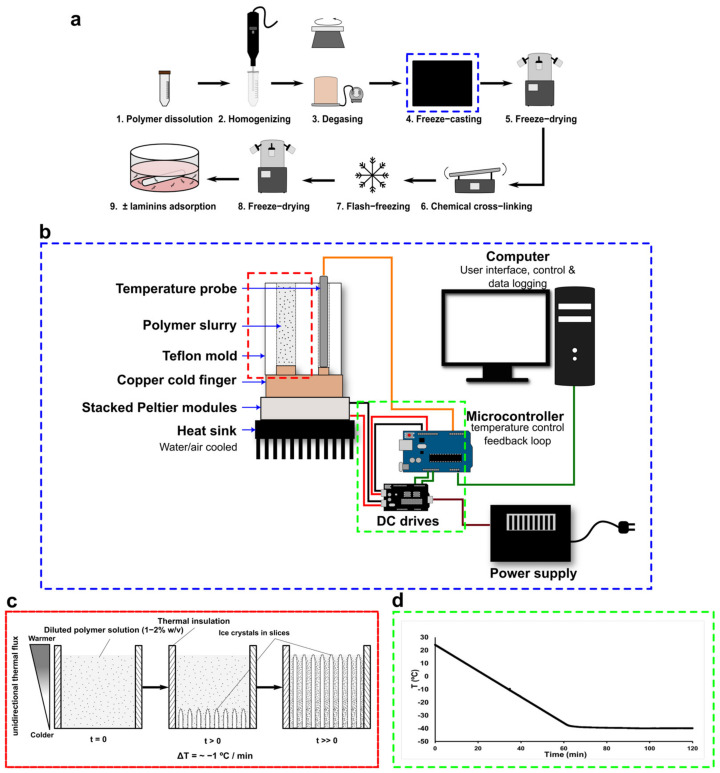
(**a**) Steps for the synthesis of anisotropic scaffolds by unidirectional freeze-casting. (**b**) Schematic showing the major components of the unidirectional freeze-casting system based on thermoelectric elements (section framed in blue in (**a**)). (**c**) Schematic showing the freeze-casting process occurring in the Teflon mold (section framed in red in (**b**)) where ice crystals in slices grow in the direction of the thermal gradient over time when subjected to a decreasing temperature ramp. (**d**) Typical temperature/time curve observed during the freeze-casting process showing the accuracy of the control system (section framed in green in (**b**)) in respect to the set points imposed by the decreasing temperature ramp. Data are logged at a frequency of 10 Hz.

The scaffolds were prepared via the unidirectional freeze-casting method (Figure 1a) using a prototype based on thermoelectric elements developed in our laboratory. The experimental setup (Figure 1b) consists of stacked Peltier modules positioned on a heatsink, the cooling sides facing up. An in-house cylindrical mold made of Teflon and having a copper base was put in direct contact with the cooling surface. Within the mold, an RTD temperature probe with a low response time (<300 ms) was positioned at the contact of the copper surface for real-time temperature acquisition. The temperature is accurately controlled via a feedback loop using a microcontroller board (Arduino Uno). The control of the system (QT user interface), real-time temperature monitoring, and data logging (10 Hz) are achieved on a personal computer linked to the microcontroller board. This prototype has the advantage of not using any solvent (e.g., liquid nitrogen, alcohol) for the freeze-casting process, is cheaper to produce, has no moving parts, and has a low lab footprint while providing highly accurate temperature control (Figure 1d). The biopolymer suspensions (collagen or coll/chit blend) were then carefully transferred into the mold using a polypropylene syringe (10 mL Luer-Lok Tip Syringe, BD) with a gauge 10 stainless steel blunt needle (Hamilton). The freeze-casting procedure (Figure 1c) involved a controlled freezing ramp of −1 °C/min (taken at the bottom of the mold) until around −40 °C and maintained constant for 90 min (Figure 1d). Afterwards, molds were placed at −80 °C for at least 2 h before being transferred to −20 °C for 2 h. Unmolded frozen preparations were then freeze-dried (4 LCs Plus, Martin Christ) for at least 12 h to obtain the cylindrical 3D porous structures. Next, scaffolds were chemically crosslinked depending on their composition to increase their strength and limit their degradation. Scaffolds made entirely of collagen were chemically crosslinked by immersion in an ethanol 75% (*v*/*v*) solution containing 6 mM EDC and 0.36 mM NHS, which was shown to be optimal for cellular interactions with collagen-based scaffold [58,59]. Moreover, coll/chit blend scaffolds were crosslinked as previously reported [44]. Briefly, coll/chit blend scaffolds were treated by immersing them in a 95% (*v*/*v*) ethanol solution containing 33 mM EDC and 6 mM NHS. All samples were immersed in their respective crosslinking solution for 2 h at room temperature before being sequentially washed in 95% (*v*/*v*), 70% (*v*/*v*), and 50% (*v*/*v*) ethanol solutions for 10 min followed by a final washing with Millipore water (3 × 10 min). The coll/chit blend scaffolds were further chemically crosslinked using a 1% (*w*/*v*) TPP solution (pH 3.3) and immersed for 2 h before being washed with Millipore water (6 × 10 min). Finally, Millipore water was added to the scaffolds before flash-freezing by immersion in liquid nitrogen and then freeze-dried for at least 12 h as described above. Dried scaffolds were stored in a desiccator for further use.

### 2.4. Scanning Electron Microscopy (SEM) Analysis

To assess the scaffold pore and channel structure, images of crosslinked scaffolds were obtained using scanning electron microscopy (SEM) (S-3000N, Hitachi, Hitachi-High Tech, Tokyo, Japan). Segments of 5 mm in length, taken from each extremity (bottom and top) and the mid-section of freeze-dried scaffolds (±crosslinked), were used for analysis. Briefly, samples taken at the top, bottom, and middle of each scaffold blend were prepared from transversal and longitudinal cuts with a surgical scalpel blade. Cut samples were then fixed onto metal stubs with conductive carbon tape and sputter-coated with a thin layer of gold-palladium using an ion-sputter (Hummer VI, Anatech USA, Sparks, NV, USA) for 1 min at 10 mA. Observations were conducted in HI-VAC mode under 5 kV acceleration and 15 μA probe current.

### 2.5. Scaffold Pore Analysis

Images of scaffold segment cross-sections (transversal cuts) taken via SEM were then used for quantitative evaluation with an image analysis routine developed in our lab and using OpenCV (Open-Source Computer Vision Library) [60]. Briefly, individual SEM images were converted into binary images through binary thresholding. Individual pores were identified by a connected component labeling approach, which is an application of the graph theory where subsets of connected components are uniquely labeled based, in our case, on the BBDT algorithm [61]. The pore count for each image was obtained through this process. The area as well as the height and width of the bounding box of each element (pore) were also extracted. The aspect ratio was defined as the ratio between the width and height of the bounding box of each pore. Pore data were then classified in a normalized discrete distribution for comparison among experimental conditions. Experiments were performed at least 3 times.

### 2.6. Evaluation of Microstructural Feature Alignment

Images of scaffold segment cross-sections (longitudinal cuts) taken via SEM were used to analyze the channel orientation quantitatively using an image analysis routine developed in our lab and using OpenCV library. Briefly, a representative region of interest (ROI) was selected, and a Sobel filter was applied to estimate the gradient in X and Y directions. The gradient was then converted pixel-wise to orientation angles and formatted on a scale ranging from 0 to 180°, where 90° stands for perfectly vertical channels and 0° or 180° perfectly horizontal channels. Orientation angles were weighted by their coherency, defined as the ratio between the difference and the sum of the tensor eigenvalues [62]. To compare each experimental condition, angle frequencies were normalized relative to the total amount of pixels in each region of interest (probability density). Experiments were performed at least 3 times.

### 2.7. Mechanical Characterization

A custom-made traction apparatus, designed to assess the mechanical properties of rat tendons under physiological conditions (37 °C, PBS 1×) [63], was used to analyze the mechanical properties of the scaffolds. Briefly, the traction apparatus is contained inside an incubator (MIR 153, Sanyo, Osaka, Japan) and is composed of a load cell (Teadea Huntleig 1022 3 kg, Vishay Precision Group, Malvern, PA, USA) and optical encoders (resolution 12.7 µm, EM1 LIN 50-6, USDigital, Vancouver, WA, USA). In the testing compartment, scaffolds were fixed at one end to a mobile anchor connected to an electromagnetic linear actuator (P01 × 23 × 80, Linmot & MagSpring, Spreitenbach, Switzerland), and at the other end, to a fixed anchor connected to the load cell. The processing of the data acquired from the load cell and the optical encoder was conducted in real-time with an FPGA (PCI-7833R, National Instruments, Austin, TX, USA) at a rate of 100 data points/s, whereas the user control and data storage passed through a LabView interface. Before the mechanical testing, scaffolds were immersed in sterile PBS 1× (pH 7.4) for 48 h at 37 °C to simulate the physiological environment. Then, the following testing regimen was applied to each sample (non-crosslinked vs. crosslinked): (1) pre-tension of samples (3 g at 1 g/s) and (2) traction test (1%/s) until end of course. Raw data (strain/force) were further processed to extract the viscoelastic modulus, maximum tensile strength, and elongation at break from stress–strain curves. Each experimental condition was tested 6 times in quadruplicate.

### 2.8. Cell Culture

Schwann cells (S16 line, CRL-2941TM) were cultivated on cell culture-treated flasks coated with PLL in DMEM, supplemented with 10% (*v*/*v*) FBS, 1% (*v*/*v*) penicillin–streptomycin. Cells were incubated at 37 °C with 5% CO_2_ atmosphere and passage by trypsinization every 2 to 3 days. Cells were used before reaching 15 passages.

### 2.9. Seeding of the Scaffolds

Scaffold segments of 5 mm in length were put in an ultra-low attachment 24-well plate (Corning, Corning, NY, USA) and sterilized by soaking them in 70% (*v*/*v*) ethanol overnight. Scaffolds were then washed 5 times using PBS 1× before half of the samples underwent laminin adsorption by incubation at 37 °C for 2 h in a 15 µg/mL laminin-PBS 1× solution. After 3 rinses with PBS, equal amounts of cells were added at one extremity of each scaffold at a density of 5 × 10^5^ cells/mL in complete growth media and incubated at 37 °C in a humidified atmosphere (5% CO_2_).

### 2.10. Cell Viability Tests and Colonization

AlamarBlue^TM^ assays were used to quantitatively measure cell viability and proliferation at the contact of scaffolds following the manufacturer’s instructions. After 24 h, 48 h, and 72 h of incubation, cell-seeded scaffolds were removed from the incubator and the growth media was replaced with DMEM without phenol red (Wisent Bioproducts, St-Bruno, QC, Canada). AlamarBlue^TM^ working solution (alamarBlue^TM^ Cell Viability Reagent, Invitrogen) was added to each sample and incubated for 2 h (37 °C, 5% CO_2_) protected from light. Then, 100 μL of supernatant was transferred to an ultra-low adhesion black 96-well plate (Greiner Bio-One, Kremsmunster, Austria) and read on a fluorescence plate reader (Safire2, TECAN, Mennedorf, Switzerland) at 560 nm excitation, 590 nm emission with a 15 nm bandwidth. Experiments were performed 5 times in triplicate.

After 72 h of incubation, live cells were differentiated from dead cells with the Live/Dead Viability/Cytotoxicity Kit (Invitrogen) following the manufacturer’s instructions. Briefly, cell-seeded scaffolds were first washed with PBS 1× and stained for 45 min at room temperature protected from light with ethidium homodimer-1 and calcein AM dissolved in PBS 1×. Automated scans were then performed on each sample at 4× magnification with an epifluorescence microscope (EVOS FL Auto, Life Technologies, Carlsbad, CA, USA). Experiments were performed 5 times in triplicate.

### 2.11. Immunostainings

After 120 h of incubation, cell-seeded scaffolds (crosslinked and laminin-coated scaffold cross-sections) were removed from the incubator. Cells were then fixed with paraformaldehyde 4% (*w*/*v*) for 20 min at room temperature and permeabilized with 0.5% (*v*/*v*) Triton^TM^ X-100 (Millipore-Sigma) in PBS 1× for 10 min. Samples were further incubated with NGS 10% (*v*/*v*) in PBS 1× for 60 min at 37 °C to block non-specific sites. Samples were then washed with PBS 1× and incubated with primary Rabbit monoclonal antibodies against S100β (E7C3A) (#90393, diluted 1:800, Cell Signaling Technology Inc., Danvers, MA, USA) overnight at 4 °C under gentle agitation. Following 3 washes with PBS 1×, samples were further incubated with Anti-rabbit IgG (H + L), F(ab′)2 Fragment Alexa Fluor^®^ 488 Conjugate (#4412, diluted 1:500, Cell Signaling Technology Inc.) and Hoechst 33342 for nucleus counterstaining (5 µg/mL) for 90 min at 37 °C in a humidified incubator (5% CO_2_) under gentle agitation. Stained cells were kept in fresh PBS 1× and visualized at magnifications of 4×, 10×, and 40× with an EVOS FL Auto epifluorescence microscope (Life Technologies, Carlsbad, CA, USA).

### 2.12. Statistical Methods

Analysis of variance (ANOVA) and post hoc tests (i.e., Tukey–Kramer’s multiple comparison test) were performed to extract meaningful statistical results using statsmodel Python Library [64]. Data from pore size analysis were analyzed using a three-way ANOVA. Data from mechanical tests were analyzed using a two-way ANOVA. Data from alamarBlue^TM^ assays were analyzed using a four-way ANOVA. All ANOVA assays were combined with a Tukey–Kramer pairwise multicomparison post hoc test. Only *p* < 0.05 was considered statistically significant (ns *p* > 0.05, * *p* < 0.05, ** *p* < 0.01, *** *p* < 0.001).

## 3. Results

### 3.1. Pore Microstructure Characterization

**Figure 2:**
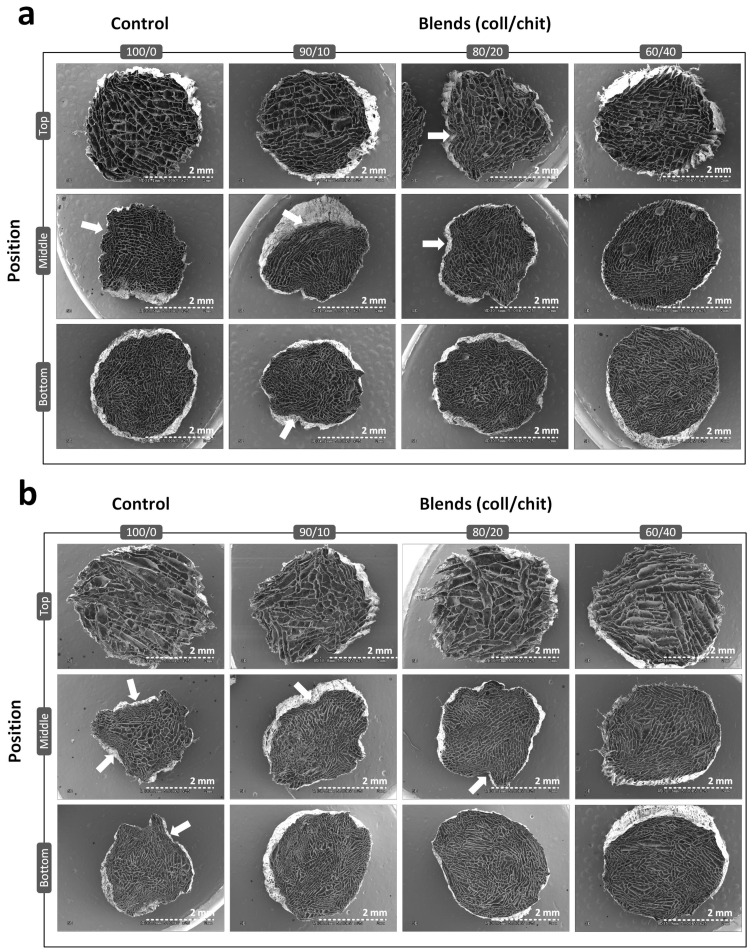
Representative SEM images showing transversal sections taken at different positions (**bottom**, **middle**, **top**) in freeze-dried scaffolds of different compositions without and with crosslinking treatment ((**a**,**b**), respectively). White arrows indicate examples of partially collapsed regions of the scaffolds after freeze-drying. Scale bars are 2 mm in length (N = 3).

**Figure 3:**
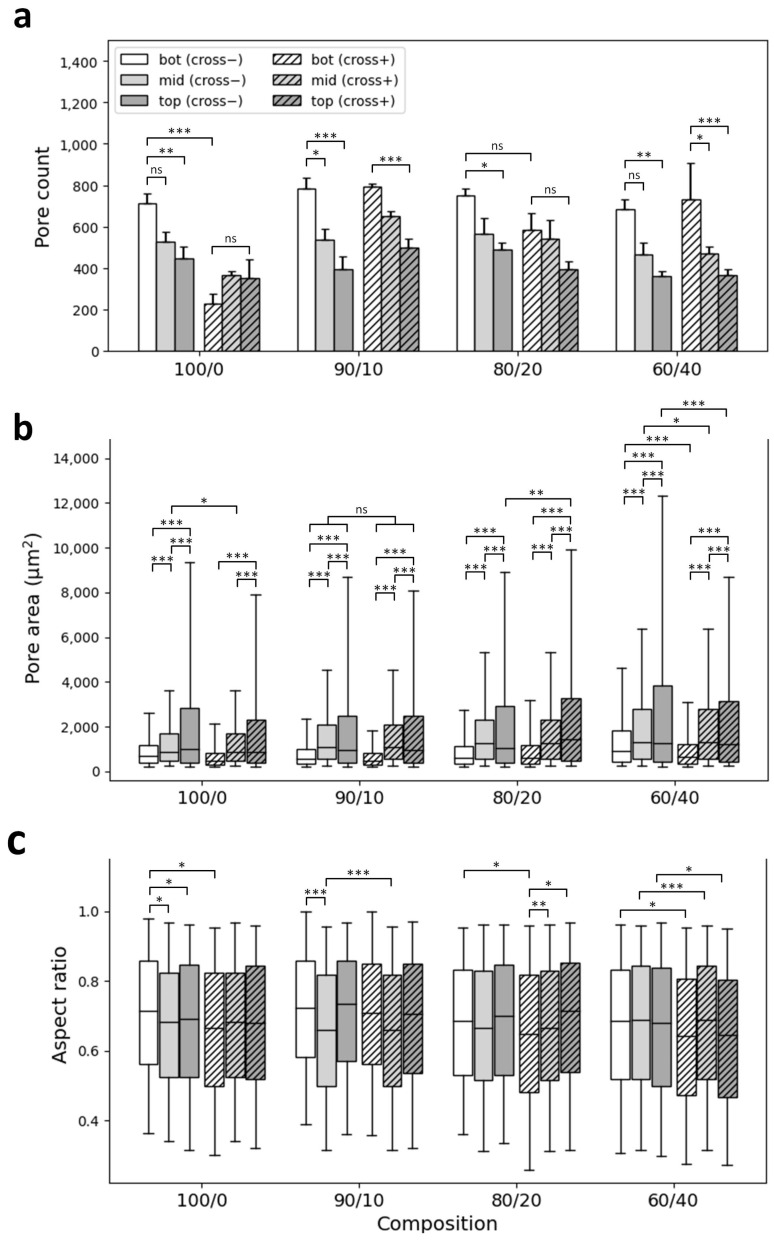
Image quantitative analysis of scaffold cross-section from SEM images. (**a**) Pore count mean ± SD values, (**b**) box plot of pore areas distributions, and (**c**) box plot of pore aspect ratio distributions for scaffolds of different compositions without (cross−) and with crosslinking (cross+) (N = 3). Only differences with *p* < 0.05 were considered statistically significant (ns *p* > 0.05, * *p* < 0.05, ** *p* < 0.01, *** *p* < 0.001).

A custom-made unidirectional freeze-casting system was used to produce vertical ice crystal nucleation in longitudinally frozen scaffolds (Figure 1). SEM images of transversal cross-sections (Figure 2) show highly porous scaffolds with a lamellar pore pattern and anisotropic structure for all compositions and conditions, typical of freeze-casting. Pore enlargement can be observed in the top section (~30 mm from the bottom) as well as a higher pore density in the bottom section. As seen in the SEM images of non-crosslinked scaffolds (Figure 2a), peripheral pores collapsed on themselves because of the freeze-drying process. This is generally observed in the middle and bottom sections of the scaffolds, where the original circular shape of the freeze-casting mold is partially lost in scaffolds and probably a consequence of thinner pore walls at these positions than at the top of the scaffold. Crosslinked scaffolds of coll/chit compositions retained their shape after crosslinking treatment, whereas the peripheral pores in the middle and bottom sections of the collagen reference collapsed further, giving the full-size scaffold a shriveled appearance. Pore count analysis (Figure 3a) correlates with the previous descriptions. For all non-crosslinked scaffold compositions, a significantly higher pore number (*p* < 0.001) is found in the bottom section compared to the middle and top sections, with the top section having a lesser number of pores. Pore count results for the crosslinked collagen reference show a significant reduction in detected pores in the bottom section compared to non-crosslinked collagen (*p* ≤ 0.001) and in the bottom section of every other composition (*p* ≤ 0.001). For the middle sections, a statistically significant difference was found between crosslinked collagen and blend 90/10 coll/chit (*p* ≤ 0.01). For the top sections, no significant difference was found between compositions and conditions. Boxplots obtained from pore area analysis (Figure 3b) show that pore area distribution is log-normal. Following pore count results, values obtained for pore areas show smaller pores in the bottom sections for all compositions and conditions. Little or no significant differences in pore area were found between non-crosslinked and crosslinked scaffolds except for blend 60/40 coll/chit, where the difference between values obtained for the bottom and top sections were statistically significant (*p* ≤ 0.001). Among non-crosslinked scaffolds, the biggest pores were obtained with blend 60/40 coll/chit, whereas blend 80/20 coll/chit yielded the biggest pores among crosslinked scaffolds. Results obtained from pore aspect ratio analysis show that pores produced have an ovoid shape. The most significant differences were found between non-crosslinked and crosslinked middle sections of blend 60/40 coll/chit as well as for blend 90/10 coll/chit (*p* ≤ 0.001).

### 3.2. Microstructural Features’ Organization and Alignment

**Figure 4:**
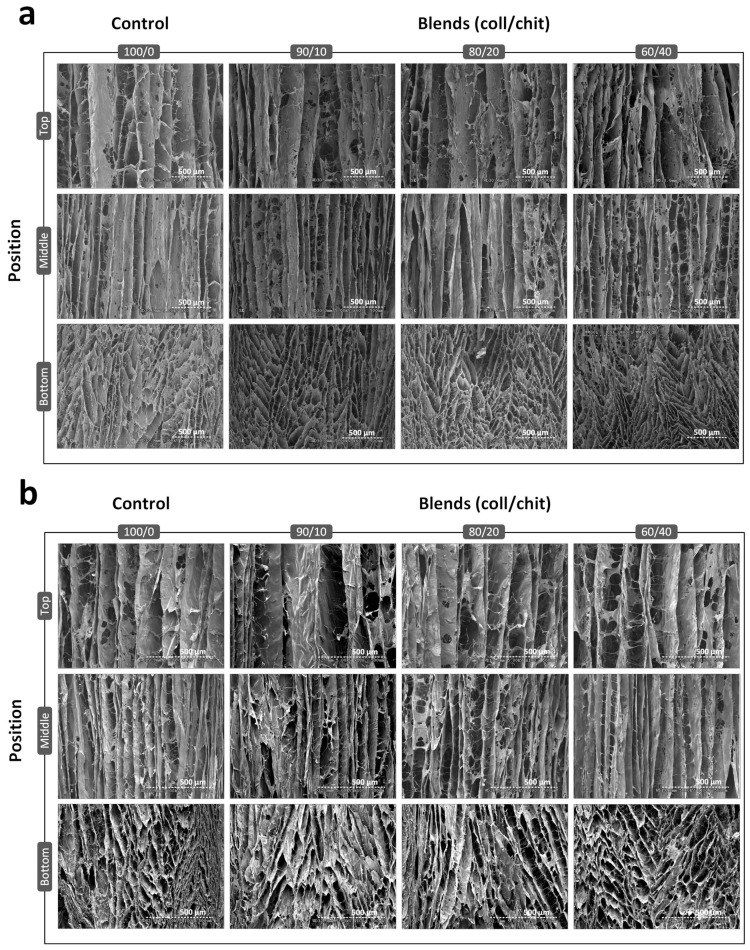
Representative SEM images showing longitudinal sections taken at different positions (**bottom**, **middle**, **top**) of freeze-dried scaffolds of different compositions without (**a**) and with (**b**) crosslinking treatment. Scale bars are 500 µm in length (N = 3).

**Figure 5:**
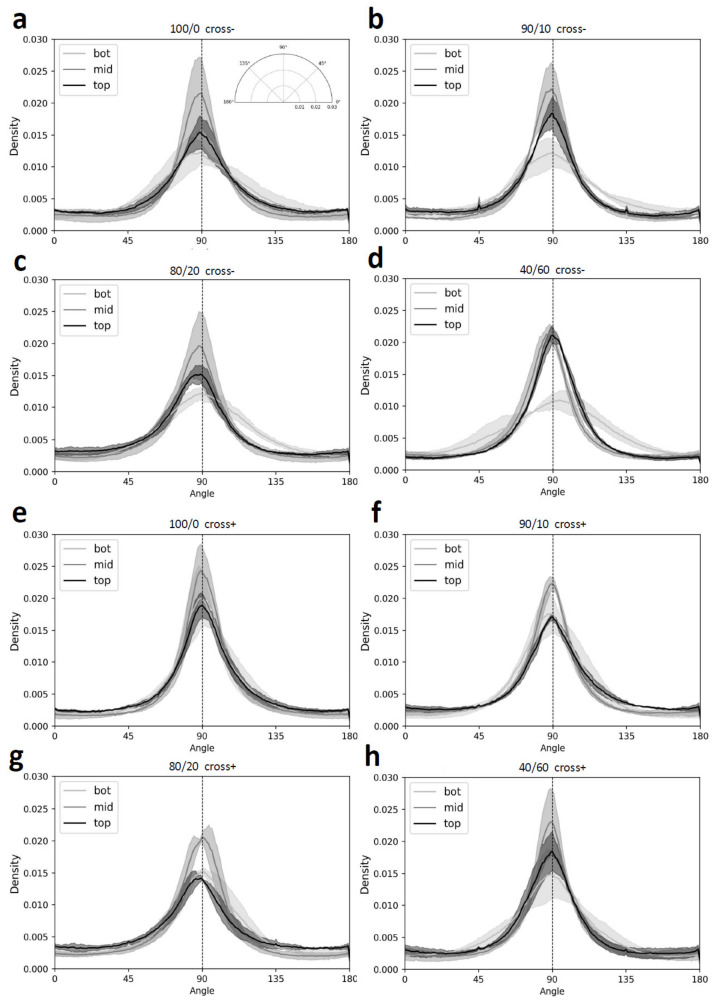
Orientation data distribution (solid lines) ± SD (corresponding color areas) for the different positions (**bottom**—**light gray**, **middle**—**gray**, **top**—**black**) determined from SEM images of longitudinal sections of freeze-dried scaffolds of different compositions without (**a**–**d**) and with crosslinking treatment (**e**–**h**) (N = 3).

SEM images were taken from longitudinally cut scaffolds from top, middle, and bottom positions (Figure 4). In the bottom images, channels initially formed in different directions and the favored orientations resulted in highly aligned parallel channels that are shown in the middle and top images. In accordance with pore area analysis, channel width increased from bottom to top. No relevant structural difference was found between compositions and conditions. Representations of image features’ orientation angle show higher densities around 90° (Figure 5). On average, maximal densities are found at 89.5 ± 1.3°, indicating that the micro-channels are aligned vertically along the length of the scaffolds. The position where channels are the most aligned is the middle section, followed by the top section and the bottom section.

### 3.3. Mechanical Properties

**Figure 6:**
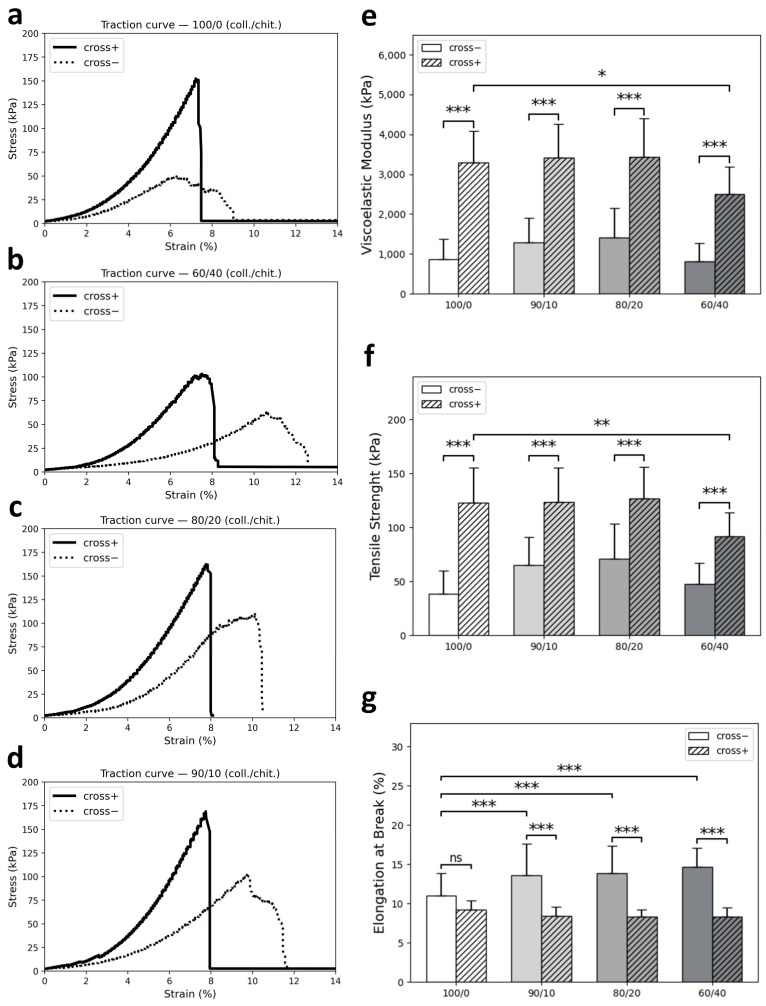
(**a**–**d**) Representative stress–strain curves obtained from traction tests of scaffolds of different compositions without (cross-) or with (cross+) crosslinking treatment (**e**–**g**) humidified in PBS 1× (48 h), mean ± SD values of extracted viscoelastic modulus, maximum tensile strength, and elongation at break (N = 24, *n* = 6). Only differences with *p* < 0.05 were considered statistically significant (ns *p* > 0.05, * *p* < 0.05, ** *p* < 0.01, *** *p* < 0.001).

Chemical crosslinkers were used to enhance the mechanical properties [44,58] of the scaffolds. Traction tests were conducted to evaluate the effects of crosslinking and composition on mechanical properties under physiological-like conditions. Data obtained were used to plot stress–strain curves from which viscoelastic moduli, maximum tensile strengths, and elongations at break were calculated (Figure 6). Typical stress–strain curves obtained from testing non-crosslinked and crosslinked scaffolds of each composition are shown in Figure 6a–d. On these curves, a toe region (low modulus region, first ⁓0–3% strain) is observed followed by a linear region (elastic part, ⁓3–6%) and a yield region (defibrillation, ⁓6–7%) and finally a failure point. Viscoelastic moduli and maximum tensile strengths results shown in Figure 6e–g demonstrated that crosslinking significantly enhanced these properties, and that this enhancement varies according to composition. When looking at mean values of viscoelastic modulus, the maximum increase is seen for the collagen reference (⁓3.8-fold), whereas 60/40 coll/chit, 80/20 coll/chit, and 90/10 coll/chit blends showed a ⁓3.1-fold, ⁓2.4-fold, and ⁓2.7-fold increase, respectively. The only statistically significant difference (*p* < 0.05) between compositions regarding viscoelastic modulus of crosslinked scaffolds is between the collagen reference (3289.3 ± 788.7 kPa) and blend 60/40 coll/chit (2499.5 ± 692.8 kPa). A similar pattern was found regarding maximum tensile strength, where crosslinking increased this property ⁓3.2-fold for collagen reference, ⁓1.9-fold for blend 60/40 coll/chit, ⁓1.8-fold for blend 80/20 coll/chit, and ⁓1.9-fold for blend 90/10 coll/chit. Here also, the only statistically significant difference (*p* ≤ 0.01) between compositions of crosslinked scaffolds is between the collagen reference (122.7 ± 32.4 kPa) and blend 60/40 coll/chit (91.8 ± 21.7 kPa). Regarding elongation at break, higher values were obtained for non-crosslinked scaffolds versus crosslinked scaffolds and weaker values obtained for the collagen reference compared to coll/chit blends in general were shown to be statistically highly significant (*p* ≤ 0.001). Furthermore, the diminishing effect of crosslinking on elongation at break was more important for coll/chit blends (*p* ≤ 0.001) compared to the collagen reference, where the difference in mean values is not shown to be statistically significant (*p* > 0.05).

### 3.4. Quantitative Analyses of Scaffold Cytocompatibility

**Figure 7:**
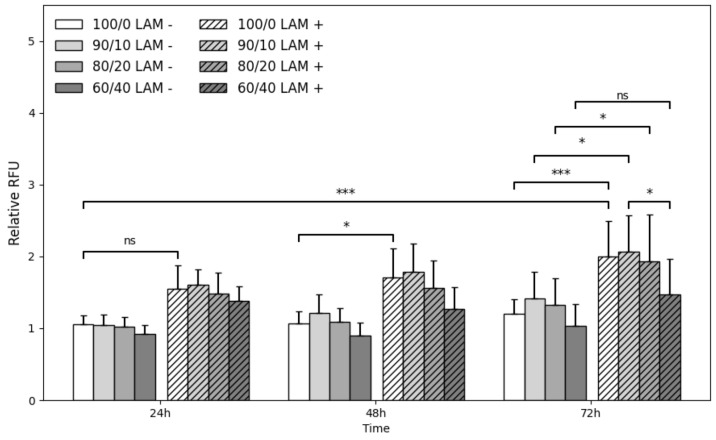
Relative RFU (mean ± SD) obtained from alamarBlue™ assays at 24 h, 48 h, and 72 h post seeding of S16 cells on crosslinked scaffold sections (L = 5 mm, Ø = 5 mm) of different compositions with added laminins (N = 15, *n* = 5). Only differences with *p* < 0.05 were considered statistically significant (ns *p* > 0.05, * *p* < 0.05, *** *p* < 0.001).

Cell viability on coll/chit blend scaffolds was evaluated using the alamarBlue^TM^ assay at different incubation times post cell seeding. Quantitative analysis demonstrated increased cell proliferation between 24 h and 72 h (Figure 7). At 24 h, apparent relative RFU differences between scaffolds without and with laminins (LAM− vs. LAM+) for all compositions was found to be not statistically significant. At the 48 h mark, the collagen reference was found to be the only scaffold composition to show a statistically significant relative RFU difference between conditions (*p* < 0.05). After 72 h post seeding, scaffolds with added laminins show higher proliferation. The difference in proliferation between conditions of the collagen reference is highly significant (*p* ≤ 0.001). Blend 60/40 coll/chit is the only composition showing no significant increase in proliferation between conditions and shows lesser proliferation compared to other compositions at every time point. Blend 60/40 coll/chit with added laminins showed significantly lower relative RFU values compared to blend 90/10 coll/chit with added laminins (*p* < 0.05). Similar results are obtained between the collagen reference and 80/20 coll/chit and 90/10 coll/chit blends. Overall, these results show that S16 cells can grow and proliferate on different compositions.

### 3.5. Cell Colonization and Viability

**Figure 8:**
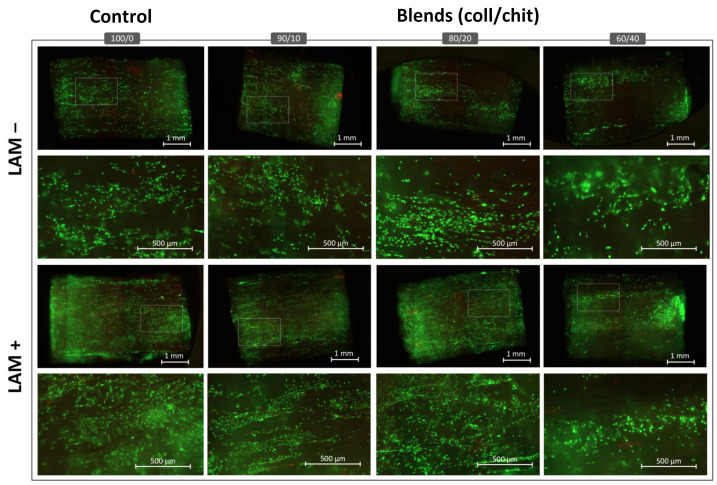
Representative scans and corresponding magnified images (representing the regions delimited by the white frames) obtained from fluorescence imaging of Live (green)/Dead (red) assays after 72 h of incubation showing attached S16 cells on crosslinked scaffolds sections (L = 5 mm, Ø= 5 mm) of different compositions with added laminins (N = 15, *n* = 5).

Fluorescence microscopy from the Live/Dead staining showed that S16 cells cultivated in direct contact with the scaffolds were mostly viable (green channel) after 72 h (Figure 8), with very few non-viable cells (red). Among the compositions, a lower number of cells was found on blend 60/40 compared to other compositions, which was congruent with the alamarBlue^TM^ assays. Cell colonization and viability were similar between the collagen reference and 80/20 coll/chit and 90/10 coll/chit blends. When laminins were added to the scaffolds, higher colonization density was found across all compositions, with blend 60/40 showing the lower number of colonized cells over the entire length of the scaffold section.

### 3.6. Immunostaining

**Figure 9:**
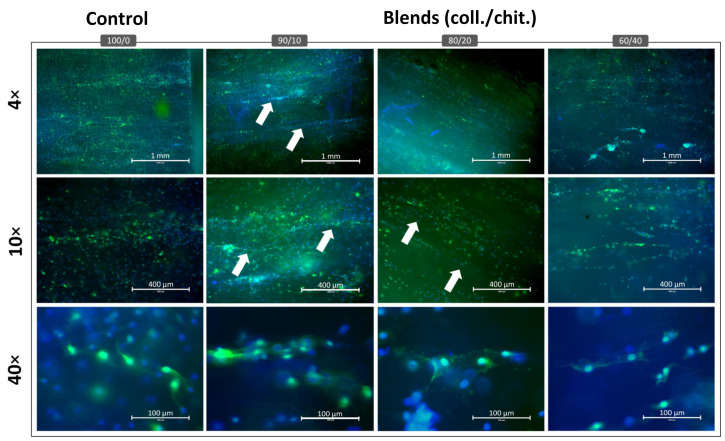
Representative images obtained from epifluorescence imaging of S16 Schwann cells cultivated at the contact of crosslinked and laminin-coated scaffold sections (L = 5 mm, Ø= 5 mm) of different compositions and immunostained against S100β (green) and nuclei (blue) after 120 h of incubation. Images are representative of 3 experiments performed in triplicate (N = 9, *n* = 3)**.** White arrows indicate cell clusters aligned parallel to the micro-channels.

Since laminin coating resulted in the best colonization density, the expression of S100β, a glial cell marker, was assessed on these scaffolds. Epifluorescence microscopy showed that S16 cells cultivated in direct contact with the scaffolds of different compositions were able to express S100β (green) after 120 h of incubation (Figure 9). On 4× magnified images, lower and less uniform fluorescent intensity and the presence of several cell clusters was observed on blend 60/40 coll/chit compared to other compositions. On 10× magnified images, 80/20 coll/chit and 90/10 coll/chit blends were the conditions showing the highest and most uniform overall fluorescence intensity in the blue (nucleus) and green (S100β) channels. These scaffold compositions had also the most visible cell alignment and organization (white arrows) within the micro-channel structures as compared with the control (100% col). As shown by 40× magnified images, S100β (green) was located mostly in the cell cytoplasm with an elongated cell body regardless of the scaffold composition. Cell morphologies produced observable alignments in the scaffold micro-channels for all conditions. However, 80/20 coll/chit and 90/10 coll/chit blends showed the most uniform cell alignments parallel to the micro-channels in the whole scaffolds, with cells expressing distinctive filipodia.

## 4. Discussion

In this study, large-size scaffolds (35 mm length, 5 mm diameter) were fabricated from various collagen and chitosan blends using a novel custom-made thermoelectric-based unidirectional freeze-casting apparatus. Scaffolds were crosslinked based on optimal parameters mentioned in other studies [44,59]. Furthermore, laminins were added to these scaffolds to enhance Schwann cell compatibility. From previous work, we know that both biopolymers used in this study (collagen, MMW chitosan 75–85% deacetylated) are proper materials for nerve regeneration [45,65]. The properties of scaffolds of 35 mm in length used as nerve guidance conduits made from combinations of these biopolymers using freeze-casting had never been reported.

### 4.1. Scaffold Structure Obtained from Thermoelectric Freeze-Casting

To our best knowledge, this is the first work reporting the use of thermoelectric freeze-casting for tissue engineering scaffolds, with only a few other publications using thermoelectric-based freeze-casting devices for the fabrication of porous ceramics meant for energy applications [35,37]. Scaffolds obtained in this study exhibited lamellar-type, highly aligned longitudinal pores displaying an overall anisotropic structure, which is meant to reproduce endoneurial channels found in peripheral nerves. Results obtained from the pore analysis show coherence between pore count and pore area (Figure 3a–b). As for the pores’ aspect ratio (Figure 3c), data are consistent for visual observations made using SEM. Many studies report the relationship between round-shaped pore diameters and their impact on the growth and linear arrangement of axons and blood vessels [66,67,68,69]. It is not clear what kind of impact lamellar-shaped pores have on blood vessel formation and axon regeneration. Thus, comparing the pore size of scaffolds from the current work with previous studies might be inappropriate, even though successful regeneration using scaffolds with lamellar shaped pores has been reported before [70]. From our results and other work, we can conclude that thermoelectric freeze-casting and solvent-based freeze-casting yield very similar structures [55,56,70,71,72,73]. Distributions of pore area and aspect ratio values are log-normal as shown before [56]. As reported in other work, we also observed that the pore area and cell wall thickness both increase with distance from the cooling source [55,56,71,72]. Additionally, we observed that the nucleation zone found at the bottom of the scaffolds contains randomly organized pores that gradually align with the freezing direction after a span of about 2 mm as seen in the bottom sections of scaffolds (Figure 4b). A similar observation was also described by Pot et al. [55]. The uneven pore size and nucleation region may be considered as the two shortcomings of the structure obtained using the current freeze-casting apparatus. Because pore area varies along the scaffolds, one might expect that the linear arrangement of the axons, glial cells, and blood vessels would progressively be less efficient with increasing pore area [74]. Nevertheless, the experimental setup used in this study is cost-effective, has a small lab footprint and requires no moving parts, and allows the fabrication of scaffolds with highly aligned micro-channels on a span of about 35 mm. The quantification of micro-channel alignment allowed us to perform a rigorous analysis instead of a simple visual appreciation.

### 4.2. Mechanical Properties under Traction

Tissue engineering constructs are required to be mechanically appropriate regarding the real biological tissue they are meant to replace. They also require high porosity in general, thus making the fabrication of such scaffolds even more challenging. As for peripheral nerve engineering, the mechanical properties of scaffolds in traction are especially important for successful regeneration. Many publications tackling mechanical properties report characteristics of scaffolds under compression [24,44,56,57,58,75] with only a few where mechanical traction tests are carried out on hydrated NGCs [26,71,76,77,78]. From another standpoint, since NGCs are meant to be used inside the living body, testing their properties under physiological conditions (37 °C, pH = 7.4) is critical. Here, a custom-made bioreactor equipped with a traction machine originally designed to test rat tendons [79] was modified to test hydrated freeze-cast scaffolds of different coll/chit compositions to obtain a variety of mechanical properties. Resulting stress–strain curves (Figure 6a–d) depicted a typical ‘J-shaped’ response under load shared with other biological tissues such as skin, ligaments, spider silk, and blood vessels [80]. This desired stress–strain relationship is associated with the orientation of fibers/polymer chains, which are at first randomly aligned and then readily orient and gradually align in the direction of an applied stress [81]. These results suggest that fibers/polymer chains in the tested scaffolds are behaving in such a way. Correlating mechanical behavior with structural analysis, a bias was observed in the fracture point of scaffolds during traction tests. Most of the scaffolds failed around the bottom section, where pore area and cell walls are smaller, indicating a higher viscoelastic modulus in the scaffold’s top sections, where pore area and cell wall thickness increase as previously mentioned. Among non-crosslinked scaffolds, coll/chit blends performed equally or better compared to the collagen reference. Higher performance regarding viscoelastic modulus and maximal tensile strength for non-crosslinked coll./chit. blends (80/20 coll/chit and 90/10 coll/chit) can be explained by intermolecular forces. Based on reports by Taravel and Domard [82,83,84], electrostatic interactions involving polyanion/polycation complexes occur between collagen and chitosan as well as hydrogen-bonding complexes. In theory, polyanion/polycation complexes are maximized at a stoichiometric ratio of 18.5% (*w*/*w*) between collagen and chitosan, which provides an explanation for why non-crosslinked high collagen blends (80/20 coll/chit and 90/10 coll/chit) performed better mechanically than non-crosslinked collagen alone. As for elongation at break in non-crosslinked scaffolds, values were higher for coll/chit blends compared to collagen alone, indicating that chitosan and collagen interactions enhanced scaffold stretchability. The crosslinking treatment of scaffolds enhanced their rigidity, increasing their viscoelastic modulus and maximal tensile strength and decreasing their elongation at break. Crosslinked coll/chit blend scaffolds performed as well as our collagen reference, with only blend 60/40 coll/chit yielding lower performance regarding viscoelastic modulus and maximal tensile strength. Despite different coll/chit ratios, crosslinked coll/chit blend scaffolds behaved the same way when looking at elongation at break results. For these reasons, it would be of interest to evaluate the effects of composition-tailored crosslinking on coll/chit blend scaffolds. Ultimately, scaffolds produced in this study would require further reinforcement to match the mechanical properties of human nerves. For example, a human acellular tibial nerve has a Young modulus of 8.19 ± 7.27 MPa, a maximum tensile strength of 8.54 ± 3.37 MPa, and an elongation break of 164 ± 34%, which all surpass the current scaffold’s properties [85].

### 4.3. Schwann Cell Colonization, Survival, Proliferation, and Functionality at the Contact of the Scaffolds

Even though collagen and chitosan are known to be materials offering good cytocompatibility, the performance of scaffolds made from different coll/chit ratios requires evaluation, especially considering the size of the scaffolds being tested. Furthermore, the next generation of tissue-engineered scaffolds will require additional elements to provide extracellular cues found in real biological tissues which are required for many cell functions [31]. Among these cues, laminins are involved in regulating cell proliferation, adhesion, and migration as well as their behavior in wound repair and play an important role in the peripheral nerve where they are involved in the myelination process of axons [52,53,86]. Previous works showed enhanced Schwann cell proliferation on scaffolds with added laminins [73,87] but such results have never been reported regarding the scaffold compositions presented in this study. Here, laminins were added via simple adsorption on crosslinked scaffolds before the addition of Schwann cells (S16). Results from alamarBlue^TM^ and Live/Dead assays confirmed the colonization/proliferation-enhancing effect of laminins on cell culture scaffolds. Here, no significant (*p* > 0.05) reduction in cell viability was observed and the highest proliferations were achieved for scaffolds with high collagen content (blends 80/20 coll/chit and 90/10 coll/chit). Scaffolds made from blend 60/40 coll/chit, which has high chitosan content, yielded the lowest results, indicating that a compromise exists between cell compatibility and mechanical properties. Lower cell viability on the 60/40 coll/chit blend could be explained by a lower number of collagen GFOGER sites available for cell adhesion [59] compared to blends with higher collagen content. Interestingly, Schwann cells were able to colonize scaffold segments across their length even though static culture conditions should limit the colonization of 3D scaffolds. Moreover, channel size did not affect the ability of Schwann cells to migrate and colonize the scaffolds.

We also assessed the expression of S100β in S16 cells seeded in laminin-coated scaffolds. S100β is a Schwann cell marker located in both the cytoplasm and nucleus and is expressed in mature myelinating and non-myelinating Schwann cells [88,89]. S100β has been associated with differentiated Schwann cells and was shown to be involved in suppressing proliferation while promoting the myelination process through SOX10 transcription factor [90]. In this study, we observed a strong expression of S100β in cells seeded in collagen-rich composite scaffolds (blends 80/20 coll/chit and 90/10 coll/chit). These very scaffold compositions also promoted a better Schwann cell organization and alignment parallel with the micro-channels, with individual cells displaying many cytoplasmic projections (filopodia). Zhou et al. [91] observed similar rat Schwann cell behavior in pure chitosan-based nerve guidance conduit having micro-channels produced from a combination of freeze-casting and electrospinning. In the normal repair process, Schwann cells organize and align themselves in structures called Büngner bands to promote axonal growth and guidance [92]. Scaffolds with micro-channels pre-seeded with Schwann cells have been shown to emulate these alignments and promote the penetration and regeneration of axons into 3D scaffolds [91,93,94] with structure and cell organization similar to what we observed, thus emphasizing the importance of material anisotropy for nerve tissue regeneration.

Overall, results regarding Schwann cell proliferation, viability, and functionality suggest that scaffolds made from coll/chit blends are good candidates for tissue engineering applications. However, further studies are now required for full-size scaffolds (≥30 mm in length).

## 5. Conclusions

In this work: a novel custom-made thermoelectric-based freeze-casting system was used to fabricate scaffolds of coll/chit blends varying in ratios from which microstructural features, mechanical properties, and Schwann cell compatibility were characterized and set against a collagen reference. Enhanced mechanical performances were obtained using a combination of crosslinking agents and cell interaction with the scaffolds, which was enhanced by adding cell adhesion proteins (laminins). Corroborating previous work [44], 80/20 coll/chit and 90/10 coll/chit blends allowed optimal mechanical properties without the use of crosslinking agents. Therefore, this study supports collagen and chitosan as valuable and interesting biomaterials for the fabrication of nerve guidance conduits as they also promote Schwann cell survival, colonization, and functionality. Cell interaction with the scaffolds was further enhanced by incorporating laminins into the scaffolds via adsorption, demonstrating a simple and efficient approach to functionalizing scaffolds. As for freeze-casting, the results presented here support the use of thermoelectric elements as a cooling strategy of choice to produce large porous scaffolds with aligned micro-channels for the reparation of bigger nerve gaps. This work also demonstrates that freeze-casting apparatuses can be greatly simplified while still attaining the desired scaffold properties. In the future, this multistep manufacturing approach should allow the production of more sophisticated scaffolds, better mimicking real peripheral nerve tissue or other biological tissues, and could be combined with other manufacturing approaches, such as 3D printing [95,96,97], to obtain more elaborated products. Moreover, other cell types, such as stem cells, should be tested on biomaterials made from 80/20 coll/chit blends.

## Data Availability

The data presented in this study are available on request from the corresponding author.
